# Prevalence and predictors of diarrhea among children under five in Ghana

**DOI:** 10.1186/s12889-023-17575-7

**Published:** 2024-01-11

**Authors:** Marian Yenupini Kombat, Sandra Boatemaa Kushitor, Edward Kofi Sutherland, Millicent Ofori Boateng, Stephen Manortey

**Affiliations:** 1 Department of Community Health, Ensign Global College, Kpong, Ghana; 2 Department of Food Science and Centre for Sustainability Transitions, Stellenbosch University, Stellenbosch, South Africa; 3 The Johns Hopkins Bloomberg School of Public Health, Baltimore, USA

**Keywords:** Child health, Childhood illness, Ghana, Diarrhea, Demographic and health survey

## Abstract

**Background:**

Globally, childhood diarrhea is a major public health concern. Despite numerous interventions that have been put in place to reduce its incidence over the years, childhood diarrhea remains a problem and is the fourth leading cause of child mortality in Ghana. This study examined the predictors of diarrhea among children under the age of five in Ghana.

**Methods:**

Data from the 2014 Ghana Demographic and Health survey, a cross-sectional survey, was used for the purpose of this study. A total of 2,547 children under the age of five were included in this study. Logistic regression analysis was performed to establish the factors associated with childhood diarrhea and ascertain explanatory variables.

**Results:**

The prevalence of diarrhea was 11.7%. Male children (13.4%) and those living in rural areas (12%), particularly in the Brong Ahafo region (17%) recorded the highest prevalence of diarrhea. Children aged 6 to 35 months of age, maternal age and education, sex of children and region of residence were the predictors of diarrhea among children under the age of five years in this study.

**Conclusion:**

To lessen the prevalence of diarrhea among children under five in Ghana, existing interventions must be evaluated in the context of the predictors identified. Based on observations deduced from this study, the Ministry of Health, Ghana Health Service and other health regulatory agencies should intensify monitoring and awareness in the various regions, particularly in the transition and savannah zones on the causes, risk factors, and methods of preventing diarrhea in children under five. Various stakeholders including government and non-governmental organizations should take into account the predictors of diarrhea identified in the design of interventions to effectively reduce morbidity and mortality associated with childhood diarrhea.

## Background

According to the World Health Organization, diarrhea is a condition that manifests as the passing of three or more loose or liquid stools per day for an individual or the passing of those stools too frequently [46]. Over the years, diarrheal disease has been a significant public health concern, especially in developing nations like those in Asia and sub-Saharan Africa that experience high morbidity and mortality rates among children under the age of five [32, 45].

Diarrhea is often classed as acute or chronic based on clinical symptoms and presentation [34]. Acute diarrhea is common and is thought to be the primary cause of illness, dehydration, and death in young children [19, 45]. On the other hand, chronic diarrhea can lead to various gastrointestinal and neurological issues that may have an impact on a child's development and growth. In order to distinguish between acute diarrhea, which typically resolves on its own, and chronic diarrhea, which may require some intervention or therapy, an increase in the frequency of defecation or stool is sometimes employed as a primary criterion [38].

Even though most regions of the world have seen a decline in the number of fatalities due to diarrhea over the years, diarrhea continues to be a major cause of disease and mortality, particularly among young children living in low- and middle-income countries [11, 31, 43]. Young children are thought to be affected by roughly 63 percent of diarrheal illnesses worldwide, with most cases occurring in low- and middle-income countries [45]. When compared to other parts of the world, Africa reports the slowest reduction rates for diarrhea and the least decrease in death rates associated with diarrhea [12]. Numerous attempts to lessen the worldwide burden of diarrheal diseases have been hampered by the lack of information on the efficacy of programs [2, 40].

In Ghana, diarrheal illness is the fourth primary cause of death among young children [11]. It explains approximately 9% of all mortality among young children (under the age of five), with an estimated 113,786 diarrhea cases recorded per year for children under five years, as at 2011 [11]. Within the same time period, about 2,318 diarrhea cases had severe dehydration and 354 died giving a case fatality rate of 0.31% [3]. When it comes to the onset and subsequent prevention of diarrhea, the difficulties of inadequate water sources, poor sanitation, poor hygiene habits, as well as the complicated roles various pathogens play, should not be overlooked [8, 13, 16]. Thus, the incidence of childhood diarrhea can be prevented and decreased by increasing the quantity and quality of water, using oral rehydration salts, breastfeeding infants during the first six months of life, and encouraging good hand hygiene [30, 47].

To address the hygiene crisis in Ghana, several organizations have developed health initiatives aimed at preventing hygiene-related illnesses in Ghana. UNICEF’s Ghana ‘IWASH’ handwashing project is one such initiative which seeks to educate school children particularly those living in rural areas on the health effects of not washing their hands. Other initiatives like the rehydration, breastfeeding and clean water projects by UNICEF in Ghana also exist to provide basic preventive and curative services [25].

Despite these initiatives, childhood diarrhea continues to be a significant public health issue in Ghana. Previous research on the prevalence and determinants of childhood diarrhea have been conducted in a few districts and health facilities in Ghana as well as at the national level [1, 3, 41, 42]. Few of such studies such as that of Tampah-Naah have highlighted maternal and child level factors associated with diarrhea among children under the age of two years in Ghana (2019). Children less than two years are considered to be more prone to infectious diseases such as diarrhea as proposed in the Tampah-Naah study. Although it has been argued that children under the age of two account for the majority of occurrences of diarrhea, it is important to take into account that all children under the age of five are equally susceptible to diarrhea [41].

In addition, Afrifa-Anane et al.’s [1] study in Ghana examined factors associated with comorbidity of diarrhea and acute respiratory infections among children under five years in Ghana. Findings from this study are useful since they concentrate on the co-occurrence of diarrhea and ARIs, the most common comorbid ailment associated with diarrhea. Examining factors that influence childhood diarrhea independently however, will be beneficial in assessing the effectiveness of interventions as there will be no competing risks, disease-disease interactions, altering the balance of risks and benefits.

This necessitates a thorough analysis of the prevalence and factors associated with childhood diarrhea to gain insight into this health problem to develop effective solutions to combat it. Therefore, this study concentrated on assessing the prevalence and determinants of diarrhea among children under the age of five years using data from the 2014 Ghana Demographic and Health Survey.

## Methods

### Study setting

Ghana is a country in Western Africa with a population of roughly 31 million people, with females making up about 50.7% of the total [22]. Ghana has a wide range of medical facilities, the bulk of which are found in urban regions. In Ghana, majority of households (92 percent) had access to an improved source of drinking water as of 2021 [23]. Roughly 18 percent of households lacked access to toilet facilities [23]. Diarrheal disease among children under five years of age in Ghana has been identified as one of the top 10 causes of morbidity and morbidity [27].

### Source of data

This study made used the 2014 Ghana Demographic and Health Survey [24]. The 2014 Ghana Demographic Health Survey employed a cross-sectional design and it is the sixth edition of the series. The data collection process used a two-stage sample design. In the first stage, Enumeration Areas (EAs) in both rural and urban areas were chosen as clusters, and in the second stage, households from the selected EAs were systematically sampled for the survey. A total of 12,831 houses were included in the sample size, with about 30 being chosen from each cluster [24]. Women with children under the age of five years were the target population selected for this study. The analysis was limited to the last child after the data was weighted. The sample size used was a subset of the entire dataset and was based on respondents who had who provided data for the experience of diarrhea of their children two weeks preceding the survey. A sample size of of 2,547 (weighted sample) and 2,635 (unweighted sample) was achieved to be used for our study.

#### Study variables

The parameters that this study measured were divided into dependent and independent variables. The dependent variable for this study was diarrheal disease**.** The GDHS asked mothers to answer the question “Did your child have diarrhea within two weeks prior to the survey?” The responses were coded as "Yes" if they had ever experienced diarrhea; otherwise, they were classified as "No. Twelve explanatory variables were examined in the study. These variables were chosen based on their conceptual applicability and their considerable association with the outcome variable, as determined by the results of previous investigations [1, 41]. The independent variables consisted of some selected socio-demographic, and environmental variables relating to the mother and child. Socio-demographic variables included age of child, sex of child, age of mother, region, educational level, place of residence, marital status, wealth index and employment status. Environmental variables included source of drinking water, type of toilet facility, and method of stool disposal.”

#### Data analysis techniques

Two layers of analysis were done on the data. Firstly, percentage was used to summarize the prevalence of diarrhea among the target population. Next, cross-tabulation was used to look at how diarrhea varied across explanatory factors. The predictor factors for the outcome variable were then determined using multivariate logistic regression models. Two models were built to examine the factors associated with childhood diarrhea. The first model examined the independent effect of child and environmental factors on childhood diarrhea. The second model was used to examine the effect of child, maternal and environmental factors on childhood diarrhea. The results were presented in the form of Adjusted odd’s ratios (AORs) with 95% confidence intervals (CIs). Statistical significance was assessed by using a *p*-value of < 0.05. Only independent variables that had a *p*-value less than 0.05 were considered for the logistic regression. STATA (version 18.0) was used to process the data. The data was weighted using the parameters provided by the GDHS before analysis. At each stage of the data analyses, a STATA survey set statement (svy prefix command) was used to produce unbiased means and accurate variance estimates. A survey design weight of v005, primary sampling unit of v021 and strata of v023 were used in the survey set statement. A multicollinearity test was conducted to test how much a predictor’s variance is explained by the other predictors.

#### Model fit and specifications

Two models were fitted and compared based on Akaike’s information criterion (AIC). A model with small AIC is the best model which predicts the data. Therefore the model with small AIC value was selected and all interpretations and inferences were made based on this model. The first model (AIC = 1893.428) consisted of only child and environmental characteristics. The second model (AIC = 1877.604) focused on how child characteristics, environmental factors and all other variables were associated with diarrhea.We reported findings from the second model because it had the smallest AIC and was the only complete model. Prior to fitting these models, the existence of multicollinearity between independent variables was examined. The variance inflation factor (VIF) test resulted in a mean VIF of 1.46 which implied variables were within the acceptable range of less than 4, indicating that there was absence of high multicollinearity between the variables (Min = 1.00, Max = 3.21, and Mean VIF = 1.46). For test of significance we used the 95% confidence interval.

#### Ethical issues

This study included participation of human subjects. The 2014 GDHS final report states that the Institutional Review Board of ICF International and the Ethical Review Committee of the Ghana Health Service examined and approved the survey protocol, which included the collection of biomarkers [21]. To minimize bias, the GDHS data were collected using questionnaires that had already been translated where communication was hampered by the use of English. Additionally, participant’s privacy and confidentiality were protected and assured during interviews. Written or verbal consent was obtained from each research subject [26].

## Results

### Background characteristics of the study population

From the children’s characteristics analyzed, 52.8% of the children were males (Table 1). The highest proportion of these children (22.7%) were aged 12 to 23 months and the least proportion (9.1%) were those aged 6 to 11 months. The majority of the children and their caregivers lived in rural areas, with about 17.5% living in the Ashanti region. For the maternal characteristics analyzed, the age structure for the mothers revealed 45.1% of them were between the ages of 30 to 39 years. Mothers aged less than 20 were the least proportion of 3.5%.

**Table 1:** Description of maternal, child and environmental related characteristics

Variable	Category	Frequency (N)	Percentage
Child's age in months	< 6 months	343	13.5
	6–11 months	231	9.1
	12–23 months	578	22.7
	24–35 months	462	18.1
	36–47 months	481	18.9
	48–59 months	453	17.8
Sex	Male	1345	52.8
	Female	1202	47.2
Residence	Urban	1173	46.1
	Rural	1374	53.9
Region	Western	257	10.1
	Central	298	11.7
	Greater Accra	400	15.7
	Volta	193	7.6
	Eastern	224	8.8
	Ashanti	446	17.5
	Brong Ahafo	225	8.9
	Northern	331	13
	Upper East	103	4.1
	Upper West	70	2.7
**Maternal characteristics**			
Mother's age	< 20 years	88	3.5
	20–29 years	1027	40.3
	30–39 years	1147	45.1
	40–49 years	285	11.2
Mother's education	No education	741	29.1
	Primary	504	19.8
	Secondary	1222	48
	Higher	80	3.1
Wealth index	Poorest	585	23
	Poorer	517	20.3
	Middle	515	20.2
	Richer	468	18.4
	Richest	462	18.2
Marital status	Never married	168	6.6
	Married	1633	64.1
	Living with partner	608	23.9
	Widowed	28	1.1
	Divorced	25	1
	No longer	86	3.4
Employment status	Unemployed	449	17.6
	Employed	2098	82.4
**Environmental characteristics**			
Source of water	Not improved	340	13.4
	Improved	2207	86.7
Type of toilet facility	Not improved	846	33.2
	Improved	1701	66.8
Method of stool disposal	Used toilet	186	7.3
	Rinsed into toilet	693	27.2
	Rinsed into drain	186	7.3
	Thrown into garbage	1240	48.7
	Buried	102	4
	Left in the open	140	5.5

Source: [21]

Regarding the level of education of mothers, the highest proportion of mothers had completed secondary education (48.0%), whilst the least proportion of mothers had completed tertiary education (3.1%). Higher proportions of mothers (82.4%) had some form of employment within a year prior to the survey, with a few being employed as at the time of the survey, whilst about 17.6% were unemployed. Regarding the marital status of mothers, 6 out of 10 of the mothers were married, and the least proportion of mothers (1.0%) were divorced. In terms of wealth index, 23.0% of the respondents were within the poorest wealth index bracket with about 18.2% in the richest wealth index bracket.

Higher proportions of the respondents had improved sources of drinking water and toilet facilities at 86.7% and 66.8%, respectively. Regarding the method of stool disposal, the highest proportion (48.7%) of households reported throwing or disposing of stool products into their garbage. The least proportion of households (4.0%) buried stool whilst about 7.3% of households had access to a toilet facility in their homes.

### Diarrhea prevalence by socio-demographic characteristics

Table 2 shows diarrhea prevalence by socio-demographic characteristics with corresponding level of significance. The overall prevalence of diarrhea among children below the age of five years was 11.7%. The majority of the children that presented with diarrhea were between the ages of 12 to 35 months. Male children and those living in rural areas, particularly in the Brong Ahafo region recorded the highest prevalence of diarrhea. Also, the highest prevalence of diarrhea was recorded among children with young mothers and mothers with no education and employment. Age of the child, sex, region of residence, mother’s age and level of education, wealth index and stool disposal had significant association with diarrhea among children under the age of five years in Ghana from the bivariate analysis.

**Table 2:** Diarrhea prevalence by socio-demographic characteristics

Variable	Category	Yes n(%)	No n (%)	Chi-square; *p*-value
Overall Diarrhea prevalence		298(11.7)	2249(88.3)	
Child's age in months	< 6 months	22(6.4)	321(93.6)	X^2^ = 75.62; *p* < 0.001
	6–11 months	26(11.3)	205(88.7)	
	12–23 months	103(17.8)	474(82.2)	
	24–35 months	81(17.5	380(82.5)	
	36–47 months	31(6.4)	451(93.6)	
	48–59 months	35(7.7)	417(92.3)	
Sex	Male	180(13.4)	1165(86.6)	X^2^ = 9.20; *p* = 0.002
	Female	118(9.7)	1084(90.3)	
Type of place of residence	Urban	131(11.2)	1042(88.8)	X^2^ = 0.01; *p* = 0.911
	Rural	167(12.2)	1206(87.8)	
Region	Western	19(7.4)	238(92.6)	X^2^ = 32.36; *p* < 0.001
	Central	25(8.4)	273(91.6)	
	Greater Accra	32(8.0)	368(92.0)	
	Volta	15(7.8)	178(92.2)	
	Eastern	31(13.8)	193(86.2)	
	Ashanti	65(14.6)	381(85.4)	
	Brong Ahafo	39(17.3)	187(82.7)	
	Northern	50(15.1)	281(84.9)	
	Upper East	11(10.7)	92(89.3)	
	Upper West	1115.7)	58(84.3)	
**Maternal characteristics**				
Mother's age	< 20 years	18(20.5)	70(79.5)	X^2^ = 11.98; *p* = 0.007
	20–29 years	120(11.7)	906(88.3)	
	30–39 years	128(11.2)	1020(88.8)	
	40–49 years	33(11.6)	252(88.4)	
Mother's education	No education	106(14.3)	635(85.7)	X^2^ = 8.45; *p* = 0.038
	Primary	65(12.9)	439(87.1)	
	Secondary	123(10.1)	1099(89.9)	
	Higher	4(5.0)	76(95.0)	
Wealth index	Poorest	78(13.3)	507(86.7)	X^2^ = 11.29; *p* = 0.023
	Poorer	70(13.5)	446(86.5)	
	Middle	73(14.2)	442(85.8)	
	Richer	40(8.5)	429(91.5)	
	Richest	37(8.0)	426(92.0)	
Marital status	Never married	26(15.5)	142(84.5)	X^2^ = 4.24; *p* = 0.515
	Married	187(11.5)	1446(88.5)	
	Living with partner	65(10.7)	543(89.3)	
	Widowed	6(21.4)	22(78.6)	
	Divorced	2(8.0)	23(92.0)	
	No longer	12(14.0)	74(86.0)	
Employment status	Unemployed	60(13.4)	389(86.6)	X^2^ = 0.19; *p* = 0.660
	Employed	239(11.4)	1859(88.6)	
**Environmental characteristics**				
Source of water	Not improved	36(10.6)	304(89.4)	X^2^ = 1.25; *p* = 0.263
	Improved	262(11.9)	1945(88.1)	
Type of toilet facility	Not improved	110(13.0)	737(87.0)	X^2^ = 0.20; *p* = 0.656
	Improved	189(11.1)	1512(88.9)	
Method of stool disposal	Used toilet	13(7.0)	173(93.0)	X^2^ = 13.21; *p* = 0.021
	Rinsed into toilet	83(12.0)	610(88.0)	
	Rinsed into drain	29(15.6)	157(84.4)	
	Thrown into garbage	134(10.8)	1106(89.2)	
	Buried	15(14.7)	87(85.3)	
	Left in the open	24(17.1)	117(82.9)	

Source: [21]

*Abbreviation*: *X*^*2*^ Chi-square

^*^Statistically significant at *P* < 0.05

### The predictors of childhood diarrhea

Table 3 indicates the results from a multivariate analysis conducted to examine the factors associated with childhood diarrhea. From the multivariate analysis run, children aged 12 to 23 months were 3.8 times more likely to have diarrhea when compared to those who were aged below 6 months (AOR = 3.78; 95% CI = 2.34–6.11). Female children were 0.3 times less likely to have diarrheal when compared to male children (AOR = 0.68; 95% CI = 0.53–0.86). Children living in the Ashanti region (AOR = 2.36; 95% CI = 1.31–4.26) were more likely to have diarrheal than their counterparts living in the Western region. Also, children who had mothers aged 40 to 49 years were less likely to be diarrheal than those who had mothers aged less than 20 years (AOR = 0.37; 95% CI = 0.18–0.74). The odds of developing diarrhea among children who had mothers with tertiary education was 0.5 times less likely when compared to children born to mothers with no education (AOR = 0.51; 95% CI = 0.20–1.34).

**Table 3:** Multivariate logistic regression

Variables	Categories	Model 1 AOR (95% CI)	Model 2 AOR (95% CI)
Child's age in months	< 6 months	Ref	Ref
	6–11 months	1.94 [1.09–3.47]^*^	1.91 [1.06–3.44]^*^
	12–23 months	3.54[2.21–5.66]^*^	3.78 [2.34–6.11]^*^
	24–35 months	3.13 [1.93–5.08]^*^	3.26 [1.99–5.35]^*^
	36–47 months	1.18 [0.68–2.14]	1.23 [0.70–2.14]
	48–59 months	1.14 [0.66–1.98]	1.16 [0.66–2.04]
Sex	Male	Ref	Ref
	Female	0.69 [0.54–0.87]^*^	0.68 [0.53–0.86]^*^
Source of water	Not improved	Ref	Ref
	Improved	1.20 [0.84–1.71]	1.25 [0.86–1.82]
Type of toilet facility	Not improved	Ref	Ref
	Improved	0.96 [0.74–1,25]	1.09 [0.80–1.50]
Method of stool disposal	Used toilet	Ref	Ref
	Rinsed into toilet	1.88 [0.97–3.65]	1.72 [0.87–3.39]
	Rinsed into drain	2.52 [1.20–5.28]^*^	1.85 [0.86–3.99]
	Thrown into garbage	1.51 [0.79–2.90]	1.23 [0.63–2.41]
	Buried	2.35 [1.08–5.09]^*^	2.20 [0.98–4.93]
	Left in the open	2.24 [1.06–4.75]^*^	1.61 [0.74–3.52]
Residence	Urban		Ref
	Rural		0.77 [0.55–1.10]
Region	Western		Ref
	Central		1.23 [0.66–2.26]
	Greater Accra		1.09 [0.53–2.24]
	Volta		0.75 [0.37–1.53]
	Eastern		1.86 [1.02–3.40]^*^
	Ashanti		2.36 [1.31–4.26]^*^
	Brong Ahafo		2.13 [1.21–3.76]^*^
	Northern		2.20 [1.19–4.09]^*^
	Upper East		1.06 [0.52–2.17]
	Upper West		2.03 [1.07–3.85]^*^
Mother's age	< 20 years		Ref
	20–29 years		0.42 [0.23–0.76]^*^
	30–39 years		0.42 [0.22–0.76]^*^
	40–49 years		0.37 [0.18–0.74]^*^
Mother's education	No education		Ref
	Primary		0.87 [0.61–1.25]
	Secondary		0.65 [0.45–0.92]^*^
	Higher		0.51 [0.20 -1.34]
Wealth index	Poorest		Ref
	Poorer		1.25 [0.85–1.84]
	Middle		1.52 [0.95–2.42]
	Richer		0.85 [0.47–1.53]
	Richest		0.75 [0.36–1.54]
Marital status	Never married		Ref
	Married		0.89 [0.51–1.56]
	Living with partner		0.84 [0.47–1.48]
	Widowed		1.79 [0.67–4.80]
	Divorced		1.09 [0.28–4.17]
	No longer		1.08 [0.46–2.53]
Employment status	Unemployed		Ref
	Employed		0.96 [0.68–1.34]

Source: [21]

*Abbreviation*: *Ref *reference category

^*^Statistically significant at *P* < 0.05

## Discussion

This study assessed the predictors of diarrheal disease among children under the age of five in Ghana based on data from the 2014 GDHS. The association between a wide range of crucial elements including various socio-demographic, economic and environmental factors and diarrhea was assessed among young children in Ghana. Children aged 6 to 35 months of age, maternal age and education, sex of children and region of residence were the predictors of diarrhea among children under the age of five years in this study.

The total prevalence of diarrhea from this study was about 11.7% which is relatively lower than the reported prevalence of diarrhea among children under five years in Ghana from the 2008 GDHS which was 19.8% [24]. Similarly, a study conducted on the trends and determinants of diarrheal disease among children under five in Ethiopia also reported a similar decrease in the prevalence of diarrhea from 2000 to 2016 [35]. Another study in Plateau state, Nigeria however established an increasing trend in the prevalence of childhood diarrhea from 2013 to 2017 [29]. The decrease in the prevalence of diarrhea in Ghana may be attributed to the improvements in sanitation over time, the rise in the number of health facilities which has facilitated access and coverage to various health services thus leading to significant improvements in interventions like nutrition and WASH and the various public health interventions that have been put in place over the years [14].

Children aged 12 to 23 months recorded a higher prevalence of diarrhea when compared to other age groups. This study's results contradicts those from a study conducted in Nairobi, Kenya, which established that most diarrhea cases were seen in children aged 6–11 months [33] and aligns with findings from a study conducted in Western Ethiopia that saw the largest peak of diarrhea amongst infants who were 12–23 months [2]. Results from our study further established there was a significant association between children aged 6 to 35 months of age and diarrhea. Children in this age group also had the greatest likelihood of experiencing diarrhea. This pattern was consistent with earlier research done in Sub-Saharan Africa [7, 39].

For instance, a study conducted in Sudan where children between 6 to 35 months of age had a higher likelihood of experiencing diarrhea than their counterparts who were above 35 months of age revealed several mechanisms, including maternal antibodies against enteric pathogens and ongoing breastfeeding, provided some protection against diarrhea in children under 6 months old, thus resulting in a lower likelihood of experiencing diarrhea in this age group [39]. Additionally, Ayuk et al.’s study [7] in Cameroon revealed the lower prevalence of diarrhea among children aged older than 35 months could be attributed to the intrinsic immunity of children within this age group that may have developed. On the other hand, it was determined that the introduction of complementary foods and modifications in dietary practices were to blame for the high prevalence of diarrhea in children aged 6–35 months.

According to our study, there was significant association between regions in the northern parts of Ghana (transition and savannah zones) and diarrhea. These findings were consistent with findings from a study conducted in Ghana [5]. This finding could be attributed to the varied performance of the rotavirus vaccine in Ghana, where the northern region of the country has seen a reduced impact from the vaccine [5]. Diarrhea is known to be one of the side effects of Rotavirus [18]. Secondly, the sparse distribution of WASH infrastructure, primarily in the transition and savannah zones, may account for this finding [4]. For instance, the proportion of households without toilets and those that practice open defecation is higher in the transition and savannah zones [36].

Our study revealed the sex of children was significantly associated with diarrhea. The odds of developing diarrhea among female children was less likely than male children. This finding corroborates results from studies conducted in Yemen, Ethiopia and Nigeria which identified that more male children were affected by diarrhea than female children [9, 20, 48]. Findings from Bahartha et al.’s [9] study was attributed to a possible variation in sampling techniques and size that might have been used in the study. In many studies, the role of social determinants of health and disease, such as sex, in this case, are often under-emphasized [28]. It is conceivable that this variation in the occurrence of diarrhea may be explained by environmental exposures that vary by sex and age. To fully comprehend the ways in which male and female exposures differ and how that affects their risk of developing diarrhea, additional investigation is needed.

Finally, the findings of this study on maternal age and education highlight the fact that mothers younger than the age of twenty and those with lower educational levels put their children at higher risk for having diarrhea, which is in line with a cross-sectional study conducted in Egypt, Zimbabwe, Uganda and India [6, 10, 15, 37]. Evidence which exists to support the impact of maternal education on children's overall living conditions, feeding practices, health amongst others might have accounted for this finding [17]. Also, our study aligns with studies conducted in Brazil that found younger mothers to be associated with a higher prevalence of diarrhea among their children. It is likely that older mothers have more experience in childcare and feeding [44].

### Strengths and limitations of this study

The use of a sizable, nationally representative dataset was the major strength of this study as it makes the findings of this study suitable to be generalized for the entire Ghanaian population. The limitation of this study was that the classification for the prevalence of diarrhea was according to the symptoms and signs reported by the mothers of children surveyed, and therefore was not validated by any medical personnel. Also, there was a possibility for recall bias since the mothers were asked about past events they might have forgotten about, leading to the acquisition of incorrect responses. Lastly, due to the cross-sectional nature of the study, all of the data used in the regression analysis were collected at the time of the survey, therefore they can only indicate statistical relationships between the predictor and dependent variables and not a cause-and-effect link.

## Conclusion and recommendations

This study aimed at establishing the predictors of diarrhea among children below the age of five years in Ghana. Children aged younger than 35 months of age, maternal age and education, sex of children and region of residence were the predictors of diarrhea among children under the age of five years identified in this study. Based on observations deduced from this study, the Ministry of Health, Ghana Health Service and other health regulatory agencies should intensify monitoring and awareness in the various regions, particularly in the transition and savannah zones on the causes, risk factors, and methods of preventing diarrhea in children under five. Also, policymakers and health practitioners should consider the predictors of diarrhea identified from this study in the design of interventions to help reduce the prevalence of diarrhea in Ghana. Finally, maternal and caregiver education has being identified as a key predictor of childhood diarrhea in Ghana, hence, female education should be encouraged, because it can improve the understanding of mothers, on how to prevent and manage diarrhea and other childhood illness, thereby improving child health.

## Data Availability

The GDHS data sets are accessible through the Measure DHS website (http://www.dhsprogram.com) by submitting an online request and describing the study's purpose. The data used to support the study findings are available from the corresponding author upon request.

## Abbreviations

AIC: Akaike Information Criterion
AOR: Adjusted Odds Ratio
BIC: Bayesian Information Criterion
CI: Confidence Interval
DHS: Demographic and Health Survey
GDHS: Ghana Demographic and Health Survey
GSS: Ghana Statistical Service
UNICEF: United Nations International Children’s Emergency Fund
VIF: Variance Inflation Factor
WASH: Water, Sanitation and Hygiene
